# Degenerate pathway for processing smile and other emotional expressions in congenital facial palsy: an hdEEG investigation

**DOI:** 10.1098/rstb.2021.0190

**Published:** 2022-11-07

**Authors:** Paola Sessa, Arianna Schiano Lomoriello, Gian Marco Duma, Giovanni Mento, Elisa De Stefani, Pier Francesco Ferrari

**Affiliations:** ^1^ Department of Developmental Psychology and Socialisation, University of Padova, Via Venezia 8, 35131 Padova, Italy; ^2^ Department of General Psychology, University of Padova, Via Venezia 8, 35129 Padova, Italy; ^3^ Padova Neuroscience Center (PNC), University of Padova, Via G. Orus 2b, 35129 Padova, Italy; ^4^ Section for Cognitive Systems, DTU Compute, Technical University of Denmark, 2800 Kongens Lyngby, Denmark; ^5^ Department of Medicine and Surgery, University of Parma, 43126 Parma, Italy; ^6^ Institut des Sciences Cognitives Marc Jeannerod, CNRS/Université Claude Bernard Lyon, 67 Boulevard Pinel, 69675 Bron, France

**Keywords:** motor simulation, sensorimotor simulation, facial palsy, Moebius syndrome, facial expressions, facial mimicry

## Abstract

Influential theoretical models argue that an internal simulation mechanism (motor or sensorimotor simulation) supports the recognition of facial expressions. However, despite numerous converging sources of evidence, recent studies testing patients with congenital facial palsy (i.e. Moebius syndrome) seem to refute these theoretical models. However, these results do not consider the principles of neuroplasticity and degeneracy that could support the involvement of an alternative neural processing pathway in these patients. In the present study, we tested healthy participants and participants with Moebius syndrome in a highly sensitive facial expression discrimination task and concomitant high-density electroencephalographic recording. The results, both at the scalp and source levels, indicate the activation of two different pathways of facial expression processing in healthy participants and participants with Moebius syndrome, compatible, respectively, with a dorsal pathway that includes premotor areas and a ventral pathway. Therefore, these results support the reactivation of sensorimotor representations of facial expressions (i.e. simulation) in healthy subjects, in the place of an alternative processing pathway in subjects with congenital facial palsy.

This article is part of the theme issue ‘Cracking the laugh code: laughter through the lens of biology, psychology and neuroscience’.

## 1. Introduction

Human faces are a major source of data that healthy individuals can extract in a split second by processing that occurs in distributed neural networks [1–3]. This ability may ultimately support reasoning on others' mental and affective states and allows shaping behavioural responses, especially during fast ongoing face-to-face interactions (e.g. [4]).

One of the most debated aspects in this field relates to the cognitive and neural mechanism(s) through which humans can assign emotional meaning to facial expressions.

The present research's primary aim was precisely to clarify the nature of these mechanisms and attempt to provide an explanatory model to account for such conflicting findings. If on the one side it is established that emotional processing recruits a constellation of perceptual, cognitive, affective, motor and somatosensory components (e.g. [5]), a more heated debate regards the theoretical position according to which the attribution of emotional meaning to others' facial expressions is ascribable to a mechanism of internal simulation that involves motor, somatosensory and limbic regions (sensorimotor simulation; [6]; embodied simulation; [7]).

According to the simulation models proposed in the literature, this simulation mechanism is considered either necessary for emotion processing [8] or rather a contributing component [6,9]. On the contrary, the detractors of simulation models consider simulation an unnecessary process for optimal facial expression recognition and deem as sufficient perceptual processing supported by perceptual learning [10–12].

### (a) Evidence in favour of simulation in facial expression processing

At least three lines of experimental evidence sustain the existence of a simulation mechanism underlying the recognition of others’ facial expressions. First, the studies that have considered the role of the observers' facial mimicry as a peripheral manifestation of the simulation mechanism provided evidence that the active mimicry manipulation by the experimenter [13–20] and mimicry deficiency in the case of some clinical conditions [21–23] are associated with an impairment of emotional expressions recognition/discrimination. Notably, when facial mimicry is blocked through a hardening gel, neural activity indexing visual working memory representations of emotional expressions is reduced in amplitude [24]. Second, neuroimaging studies provided convincing evidence of overlapping brain regions, including premotor, somatosensory, and gustatory cortices, involved in the production and the observation of emotional expressions [25–29]. Third, a cogent body of evidence comes from studies in both patients with brain lesions critical for the simulation accounts [30] and healthy individuals subjected to virtual lesions through transcranial magnetic stimulation [31,32]. This latter line of evidence strongly supports the role of somatosensory, motor and premotor regions in facial expression processing.

Even stronger support for the role of embodiment in emotional processing comes from a recent functional magnetic resonance (fMRI) study by Volynets and colleagues [33]. These authors used a statistical Bayesian pattern recognition technique demonstrating how executed and observed video-clipped facial expressions of joy, anger and disgust are associated with discrete neural signatures in the somatomotor system. The common neural bases of facial expressions observation and production included motor strip, supplementary motor area (SMA), somatosensory cortices, anterior cingulate cortex (ACC), amygdala, parts of the orbitofrontal cortex (OFC) and temporal pole. Notably, specific patterns of activation within the motor strip, SMA and somatosensory cortices could reliably predict which facial expression had been observed or produced by the participant. These results prove that the recruitment of motor and somatosensory regions during facial expression processing is a marker of the recovery of expression-specific sensorimotor representations (but see also [34]).

### (b) Evidence against simulation in facial expression processing

Although this set of studies, and in particular those that have employed the perturbational approach [31,32], supports a causal role of the recruitment of motor and somatosensory regions in emotion recognition, other experimental evidence seems to challenge this role dramatically, thus making the potential contribution of sensorimotor representations’ reactivation in facial expression processing less clear. A particularly relevant source of divergent evidence comes from two studies in patients with congenital facial palsy, specifically with Moebius syndrome (MBS; [35,36]).

MBS is an extremely rare congenital non-progressive condition mainly characterized by facial paralysis. Given the rarity of the syndrome, only a few studies have investigated emotion recognition in MBS patients, none using neuroimaging techniques, and, further, they present inconsistent results (for a critical review, see [21]). Nonetheless, based on the sensorimotor simulation models, one would expect that even a partial and congenital inability to recruit the facial muscles should somehow limit facial mimicry and the possibility of reactivating expression-specific sensorimotor representations. According to detractors of the simulation mechanism, evidence that these patients may perform normotypical recognition/discrimination would offer a convincing refutation of simulation models.

Using a single-case analysis approach, Vannuscorps *et al.* [36] tested 11 MBS participants in a series of eight behavioural experiments. Most of the focus was on two MBS participants who achieved normotypical behavioural performance despite their severe facial palsy, leading the authors to conclude that motor simulation is unnecessary for facial emotion recognition.^1^ Despite the seemingly flawless conclusion, these results are not necessarily antagonist to the simulation account. Firstly, when dealing with congenital and acquired nervous system disorders, it is mandatory to consider crucial variables such as neuroplasticity [37] and degeneracy, a concept introduced by Edelman indicating ‘the ability of elements that are structurally different to perform the same function or yield the same output’ [38–40]. Demonstrating that (some) individuals with congenital facial paralysis show normotypical behavioural outputs in facial expressions recognition tasks does not disprove simulation in healthy individuals, as alternative processing pathways (in light of neuroplasticity and degeneracy) may have been established in MBS individuals. Thus, neuroimaging investigations are mandatory to disclose the unfolding of facial expression processing in both MBS and control participants and, possibly, to reveal the recruitment of degenerate pathways. Secondly, previous studies have suggested that visual matching tasks, rather than labelling tasks, are more appropriate to unveil the role of sensorimotor simulation and facial mimicry in emotion processing (e.g. [20]). This observation is in line with the evidence supporting the reactivation of expression-specific sensorimotor representations during the processing of facial expressions [33]. Altogether, these observations lead to the hypothesis that simulation might be necessary to precisely characterize—from the perceiver's perspective—even subtle instances of others' facial expressions (see [24]). In this vein, very sensitive tasks are required to detect possible subtle processing deficits (in terms of accuracy and/or reaction times) in MBS subjects, and finely tuned techniques are needed to test the hypothesis of degenerate pathway activation in MBS subjects.

### (c) The present investigation

We implemented a very sensitive emotion discrimination task and the simultaneous recording of neural activity using high-density (128 sensors) electroencephalography (i.e. hdEEG). We aimed to test whether neural activity (in terms of both event-related potentials and source activations) compatible with sensorimotor simulation is observed in healthy individuals and whether neural activity, compatible with an alternative network likely of a compensative nature, is observed in MBS individuals. To this aim, seven MBS participants and seven Control participants (matched for age, gender, ethnicity and level of education) performed a subtle discrimination task of both facial expressions and equally challenging control non-face stimuli (i.e. animal shapes). In addition, using the hdEEG technique provided us with an unrivaled online window (compared to previous behavioural investigations) on the possible degenerate/compensatory processing pathway in MBS individuals.

We opted for two methodological/analytical choices. First, all the results we present are based on contrasts between brain activity (event-related potentials and source activations) elicited by instances of facial expressions randomly selected from morphing continua and brain activity elicited by instances of animal shapes randomly selected from morphing continua. This approach allowed us to isolate face/facial expression-sensitive neural activity in both Control and Moebius participants, thus excluding possible confounding factors related to a general deficiency in visual processing and object recognition in MBS (i.e. not facial expression-specific). We considered a control task that did not include facial identity and/or morphing of facial identities more appropriate since several authors have questioned the dissociability between facial expressions and identity processing domains (see, e.g. [41]).

Second, to deal with the multiple comparison problem and the Type I error, which are particularly relevant for large spatio-temporal datasets like those produced by electroencephalographic research, we opted for the state-of-the-art cluster-based permutation approach [42,43].

Using the contrastive approach reported above (facial expressions vs. animal shapes), we expected to observe in Control participants differences in the neural processing between the two stimuli categories (facial expressions versus animal shapes) from early stages, encompassing the P1 and the N170 [44,45] event-related potentials, with the involvement of regions crucial for simulation models, such as the premotor cortex and the inferior frontal gyrus (in particular the pars opercularis). Concerning MBS participants, we expected to observe facial expression processing relying on regions associated with visual and/or conceptual-semantic systems as a marker of compensatory strategies linked with degeneracy/neuroplasticity. Beyond the possible mechanisms hypothesized as underpinning compensation (particularly following brain lesions), two criteria are mandatory to define a particular brain activity as compensatory. First, it must occur in association with correct behavioural performance; and second, it must occur under demands precisely attributable to the damaged/altered region or network (e.g. [46]). In the present context, we will be allowed to define ‘compensatory’ neural activity selectively observable in MBS participants if their behavioural performance is comparable to that of control participants.

## 2. Methods

### (a) Participants

Data were collected from 15 adult participants. Seven MBS participants (MBS group: MBS 4 females and 3 males, M age = 40,43 years; s.d. = 11,03) were recruited through the Italian Association of Moebius Syndrome (AISMo) and the Operative Unit of Maxillo-Facial Surgery Head and Neck Department of the University of Parma. Data collection was conducted at the University of Padua. In table 1, demographic data and clinical information concerning all participants with MBS are reported. The inclusion criteria for adults with MBS were a certified diagnosis of unilateral or bilateral facial paralysis [47]. Exclusion criteria were (1) the presence of congenital limb malformations and (2) the presence of any psychiatric or physical illness at the time of participation. Eight healthy controls were recruited to match in terms of age, gender, ethnicity and level of education with the MBS group. One control participant was discarded for technical issues during data collection; thus, 14 participants were included in the final sample. The sample size was determined through a power analysis using data simulation for cluster-based permutation tests [48]. This analysis revealed that a sample size of five participants was sufficient to obtain power of at least 80% to detect a difference in event-related potentials (ERP) data between two conditions (face versus non-face stimuli) for a within-subject design. All participants reported normal or corrected to normal vision using lenses or glasses and did not have intellectual disabilities or other psychological or neuropsychological disorders at the time of the test. All participants gave their informed written consent after a full explanation of the procedure under the Declaration of Helsinki. The study was approved by the Ethics Committee of the University of Padova (Protocol no. 2855).

**Table 1.** Demographic data and clinical information for MBS participants

participant	age	gender	cranial nerves involved	disfunction
MBS1	54	M	abducens nerve (VI); facial nerve (VII)	no lateral eye movements; facial palsy
MBS2	57	M	abducens nerve (VI); facial nerve (VII)	no lateral eye movements; facial palsy
MBS3	38	M	abducens nerve (VI); facial nerve (VII)	no lateral eye movements; facial palsy
MBS4	25	F	facial nerve (VII)	facial palsy
MBS5	65	F	abducens nerve (VI); facial nerve (VII)	no lateral eye movements; facial palsy
MBS6	39	F	abducens nerve (VI); facial nerve (VII)	no lateral eye movements; facial palsy
MBS7	34	F	abducens nerve (VI); facial nerve (VII)	no lateral eye movements; facial palsy

### (b) Stimuli

The stimuli were 11 coloured digital pictures (i.e. face and non-face, and animal stimuli) for each morph continuum. The stimuli can be viewed at the following link of the Open Science Framework repository: osf.io/krpfb [49]. We adopted the stimuli developed by Niedenthal *et al.* [50] and used in previous studies [18,20]. The face stimuli consisted of images of a female model expressing morphed combinations of two emotions continua; sadness–anger and happiness–disgust, while the non-face control images were selected from a morph of a horse and a cow that had maximally similar postures. The face continuum began at 100% sad expression and 0% angry expression and transitioned in 20% increments to 0% sad expression and 100% angry expression. All images were generated by cropping the face region, excluding the hair, then they were resized to subtend a visual angle between 10 and 12 degrees.

### (c) Experimental procedure

Stimuli were presented on a 17 inch monitor at a resolution of 1280 × 1024 pixels. Participants were seated comfortably in a chair at a viewing distance of around 60 cm from the monitor. Each trial began with a fixation cross of 500 ms that remained in the centre of the screen throughout the trial. Then a target image appeared, presented for 750 ms. The target was a stimulus extracted from one of the three different morphing continua.

**Figure 1.**
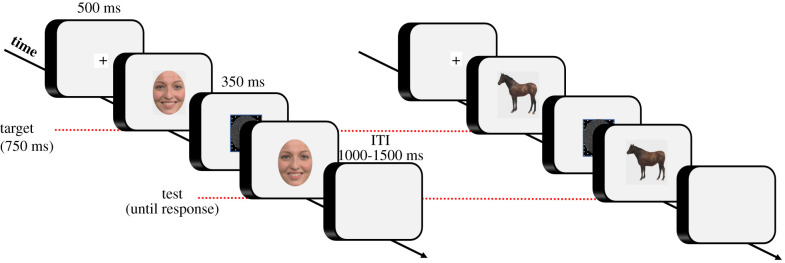
Schematic illustration of the sequence of events in two experimental trials, with facial expressions on the left, and with animals on the right (ITI, inter trial interval). (Online version in colour.)

The target was then replaced by a noise mask blank screen with a duration of 300 ms to limit the use of mnemonic strategies (see [20]), followed by a test image. The test image was extracted from the same morphing continuum as the target (figure 1). Participants were instructed to maintain their gaze on the fixation cross throughout the trial. The task was to compare the target with the test image and to indicate if the test image matched the target or not (by pressing ‘F’/‘J’ keys on the keyboard; order counterbalanced across participants). The target and image were identical in 50% of the trials. In the remaining 50% of the trials, the test image was a different stimulus from the same continuum. For example, if the target was a facial expression from the happy–disgust continuum when a change occurred, the test image was a different facial expression from the same happiness–disgust continuum. The responses had to be given without any time pressure. Following the participant's response, a variable interval of 1000–1500 ms (in 100 ms steps) elapsed before the presentation of the fixation cross, indicating the beginning of the subsequent trial.

Participants performed eight practice trials for the control animal condition, eight for the anger–sad and eight for the happiness–disgust facial expressions condition (counterbalanced order across participants) in separate blocks of trials. The trial order was randomized across participants and was separated into three blocks for each continuum with self-paced breaks every 20 trials. Participants performed 192 trials for each continuum. The total number of trials was 540. E-prime 2 software (Psychology Software Tools, Pittsburgh, USA) was used to create and administer the experiment.

### (d) Behavioural analysis

We tested our hypotheses using accuracy and reaction times (RTs) as response variables. We considered only RTs in correct trials. We analysed the effects on response accuracy and RTs by setting a single factor within-subject experimental design that we tested through generalized linear mixed-effect models (GLMMs). We defined two separate GLMMs for RTs and accuracy for each of the two groups (i.e. MBS and control) separately. Type of stimulus (i.e. Animal versus Face) was considered as a within-subject fixed factor. We set random intercept models, with participants as the clustering variable. Ninety-five percent confidence intervals (CIs) of the mean differences between conditions are reported in squared brackets. We used the R statistical software [51] to run statistical analyses, using the following packages: lme4 [52] to test the GLMMs, emmeans [53] to test multiple comparisons, and car [54] to estimate *p*-values, which were adjusted with the false discovery rate correction [55]. We reported confidence intervals (set at 99%), defined only for *t*-tests, and referred to the difference of means (i.e. Mdiff; as suggested by [56]). We also ran two separated models for RTs and accuracy, testing the interaction between Type of stimulus and Group (i.e. MBS and Control), settled as within-subject fixed factors. Results from these models are reported at the OSF repository link [49].

### (e) Electroencephalography recordings

We used a Geodesic hdEEG System (EGI GES-300) with a pre-cabled 128-channel HydroCel Geodesic Sensor Net (HCGSN-128) and electrical reference to the vertex. EEG data were recorded during the entire experiment. The sampling rate was 500 Hz. The impedance was kept below 60 kΩ for each sensor. To reduce signal contamination, participants were instructed to limit eye blinks and eye movements as much as possible during trials.

### (f) Electroencephalography pre-processing

Signal preprocessing was performed through EEGLAB 14.1.2b [57]. The continuous EEG signal was first downsampled at 250 Hz and then bandpass-filtered (0.1–45 Hz) using a Hamming windowed sinc finite impulse response filter (filter order = 8250). The signal was successively epoched between −500 and 1500 ms from the target onset. Epochs related to trials containing premature responses were rejected. Epoched data were subjected to an automated bad-channel and artefact detection algorithm using the TBT plugin [58] implemented in EEGLAB. This algorithm identified the channels that exceeded a differential average amplitude of 250 µV and marked those channels for rejection. Channels marked as bad on more than 30% of all epochs were excluded. Epochs having more than 10 bad channels were also excluded. Successively, we automatically detected possible flat channels with the Trimoutlier EEGLAB plugin, with the lower bound of 1 µV. Data cleaning was performed employing an independent component analysis [59], using the Infomax algorithm [60] implemented in EEGLAB. The independent components (ICs) were automatically labelled using the IClabel plugin [61]. ICs labelled as eye, heart, muscle, line noise or channel noise with greater than 70% confidence were marked as artefactual and rejected. The remaining components were then projected back to the electrode space to obtain cleaner EEG epochs. Finally, bad channels were reconstructed with the spherical spline interpolation method [62,63]. The data were then re-referenced to the average of all electrodes, and baseline correction was applied by subtracting the mean signal amplitude in the pre-stimulus interval. Epoched data were imported in Brainstorm [64] to generate the individual average for each electrode site and experimental condition. To isolate specific face-sensitive responses, we first subtracted the electrical activity generated by animals from that generated from faces. We used the global field power (GFP, [65], i.e. the sum of the square of all the sensors at each time point) of the differential waveforms plotted as a function of time, and the occurrence times of GFP maxima to determine the latencies of each evoked potential component for the two groups separately (±100 ms around the GFP maxima). We employed this approach because crucial differences between controls and MBS participants could be likely observed in terms of the latencies of the components sensitive to the processing of faces compared to non-face stimuli.

### (g) Cortical source modelling

Baseline-corrected epochs were imported in Brainstorm [64] to model their cortical generators. We used the ICBM152 anatomical template to approximate the individual anatomy of each participant [66]. Co-registration of EEG electrodes' position was performed via Brainstorm, projecting the digitized EEG sensor positions GSN Hydrocel 128 E1 available in Brainstorm on the head surface. We then derived an EEG forward model using the three-layer boundary element method (BEM) from OpenMEEG implemented as a Brainstorm routine [67,68]. The source space was constrained to the cortex and modelled as a grid of 15.002 orthogonal current dipole triplets. We used sLORETA as a source model with Brainstorm's default parameter settings. The empirical noise covariance model was obtained from the average of ERP baseline signals. The sources were projected to the standard anatomical template (MNI), and their activity was transformed in Z scores relative to the baseline. Finally, a spatial smooth with a 3 mm full width at half maximum was applied to each source.

### (h) Electroencephalography statistical analysis

We applied a whole-scalp analysis approach at all electrode sites using a paired *t*-test (*α* = 0.05) permutation approach to control the family-wise error rate [69]. A similar technique was employed in previous ERP studies [70–74]. To control for the Type I error, we performed 5000 Monte-Carlo permutations and applied cluster-based correction over all 128 electrode locations and including all the timepoints within the temporal windows (i.e. not averaged) using the Fieldtrip functions [75], accessible via Brainstorm [64].

Finally, concerning the source statistic, a one-tailed 1000 Monte-Carlo permutation paired *t*-test was run over the mean amplitude of the Z-scored maps, in the same (averaged in time) windows of the ERPs components.

## 3. Results

### (a) Behavioural results

**Figure 2.**
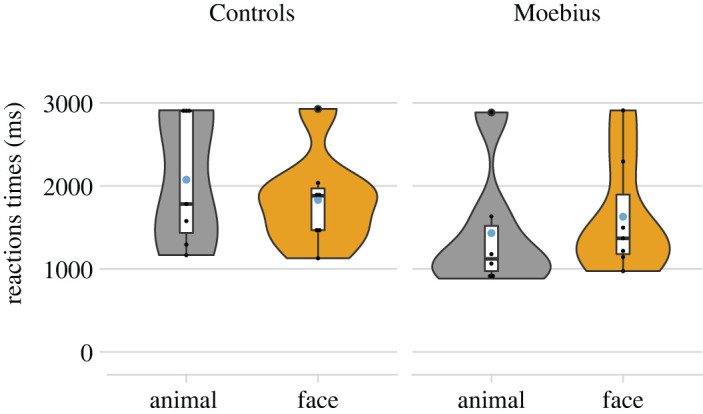
The figure shows the violin and box plot of the single-subject data for mean RTs for Control and MBS groups, separately. Light blue dots represent the mean of RTs for each type of stimulus. (Online version in colour.)

We did not observe any significant effects at the accuracy level. For RTs, we observed a statistically significant effect of the Type of stimulus both for the control ($\chi_{\left(1\right)}^{2}$ = 83.71; *p* < 0.001) and for the MBS group ($\chi_{\left(1\right)}^{2}$ = 19.12; *p* < 0.001). The two groups showed an opposite pattern (see figure 2); Controls were faster in trials with faces than animals (*t* = −9.10; *p* < 0.001, Mdiff = −172,58, 95% CI [−204,23, −132,22]), while MBS were faster with animals than faces (*t* = −4.37; *p* < 0.001, Mdiff = 46,58, 95% CI [31,22, 81,99]).

### (b) Electroencephalography results

GFP maxima of the difference waveforms (i.e. faces minus animals) indicated two different time-windows of face sensitivity for MBS and Control group. Two different peaks were identified for each group: for the Controls, from 112 to 152 ms and from 168 to 208 ms, compatible with a P1 and a N170 effect, respectively, while for the MBS, two later time-windows were identified, i.e. from 180 to 256 and from 256 to 332 ms.

**Figure 3.**
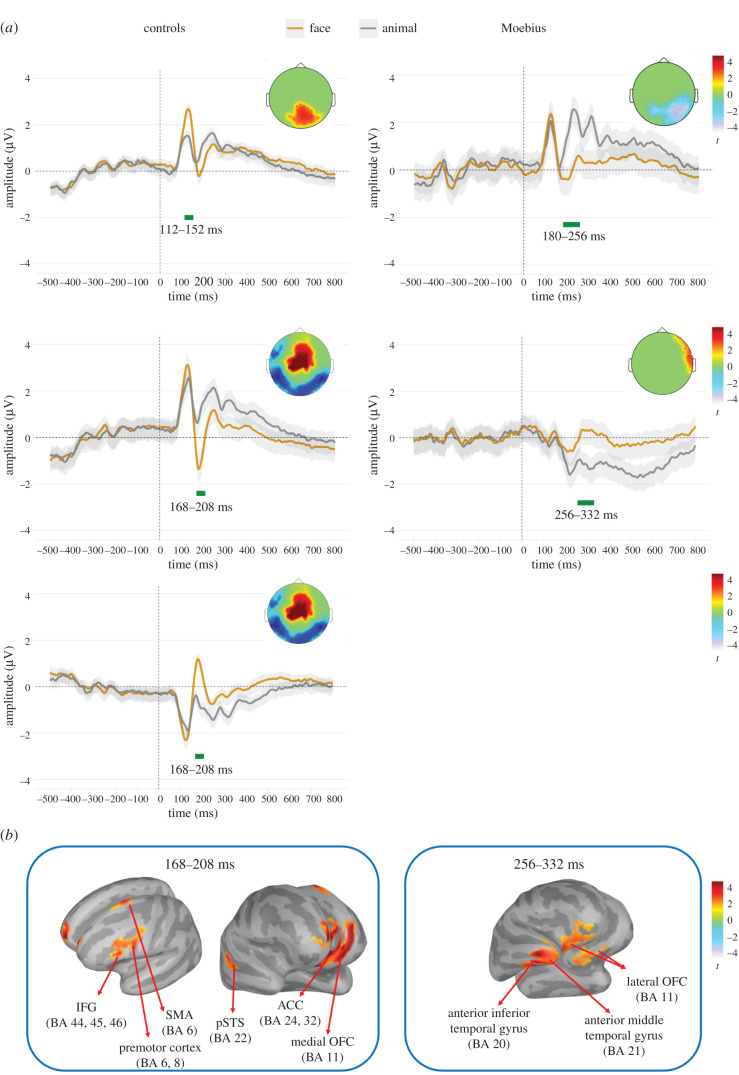
(*a*) In the top part of each plot, the images represent the statistically significant electrodes (*p* < 0.05) in each time-window derived from the cluster-based permutation analysis, separated for the two groups: the control on the left and the MBS on the right. Their colours (e.g. reddish or bluish) depend on the direction of the *t*-test. The ERPs below each scalp map show each cluster's time series for the two contrasted conditions. (*b*) The statistical difference of the source maps comparing face and animal post to the test-image stimulus activity, separated for controls (168–208 ms) and MBS (256–332 ms). Significant clusters (*p* < 0.05) are reported on a template cortex smoothed at 100%. The left panel shows a more significant activity for facial expressions than animals in the Control group in: IFG, inferior frontal gyrus (Brodmann areas 44, 45 and 46); premotor cortex (Brodmann area 6); ACC, anterior cingulate cortex; the medial OFC, orbitofrontal cortex (Brodmann area 11); and the pSTS, posterior superior temporal sulcus (Brodmann area 22). The right panel shows the greater activity for faces in the MBS group in: OFC, orbitofrontal cortex (Brodmann area 11); anterior inferior gyrus (Brodmann area 20) and middle temporal gyrus (Brodmann area 21). (Online version in colour.)

In the first time-window compatible with the P1 time range and scalp distribution (112–152 ms), the Control group presented a marginally significant positive cluster of electrodes (*p* = 0.051; cluster size = 203; cluster statistic = 604), showing a trend toward a larger amplitude in response to faces as compared to animals. The permutation analysis in the second time-window (168–208 ms) revealed a significant positive cluster of fronto-central electrodes (*p* < 0.0001; cluster size = 372; cluster statistic = 1394) and a negative cluster of occipital electrodes (*p* = 0.048; cluster size = 250; cluster statistic = −785), compatible with the N170 component (see figure 3*a*).

In the first selected time-window (180–256 ms), the MBS group presented a significant negative cluster of occipital electrodes (*p* = 0.005; cluster size = 226; cluster statistic = −714), while the second time-window (256–332 ms) revealed a positive right fronto-central cluster of electrodes (*p* < 0.022; cluster size = 74; cluster statistic = 223).

To estimate face-sensitive areas, we performed one-tailed permutations at the level of the sources by comparing the activity elicited from faces to the one from animals in the averaged late time-window including anterior clusters (Controls: 168–208 ms; MBS: 256–332 ms; *p <* 0.05) as we were particularly interested in anterior sources activation.

In the Control group, the source statistic revealed the engagement of the left IFG (pars opercularis, orbitalis and triangularis) and premotor cortex and SMA, as well as the left anterior cingulate cortex. Further, we also found a higher activation for facial expressions compared to animals in the right posterior superior temporal sulcus (pSTS) and the left medial OFC (see figure 3*b*, left panel).

The same analysis for the MBS group revealed a stronger activation for facial expressions than animals in the right anterior middle and inferior temporal gyri, the right temporal pole, and the lateral OFC bilaterally (see figure 3*b*, right panel).

## 4. Discussion

In the face of several sources of evidence supporting the hypothesis of a functional reactivation of sensorimotor representations in emotional expression recognition [25–28,30,33] some diverging evidence seems conclusive in holding that this ability does not require sensorimotor simulation.

A particularly relevant source of opposing evidence is based on normotypical abilities in facial expression recognition tasks shown by some individuals with MBS [35,36]. This evidence has been interpreted as supporting the sufficiency of a visual analysis system jointly with a perceptual learning mechanism as underlying emotion recognition. Still, these conclusions are not unquestionable and do not conclusively disprove sensorimotor simulation models.

Firstly, the tasks employed in these previous studies on MBS individuals used very high-intensity expressions and/or required them to label the expressions and, therefore, could have been not appropriately sensitive to reveal subtle deficits. Secondly, when dealing with congenital and acquired nervous system disorders, it is mandatory to consider compensatory and neuroplasticity mechanisms, likely leading to the development of alternative network(s) for facial expression recognition. Thirdly, some simulation models [6] implicitly acknowledge the brain mechanism of degeneracy [39,40] and conceive the involvement of the sensorimotor system as an important contribution within a complex network in which different nodes process partially overlapping aspects of the same type of information. From these considerations derives the need for particularly sensitive techniques and paradigms to detect whether and how emotional recognition is implemented in the brain through an alternative neural pathway in the case of the absence/deficiency of a contribution from sensorimotor regions.

In brief, these previous studies do not directly inform us on the neurocognitive functioning in healthy subjects, nor do they inform us about possible alternative compensatory networks in MBS individuals.

In the present investigation, employing a highly sensitive emotion recognition task that included control stimuli (i.e. animal shapes) and the use of hdEEG, we exactly pursued the aim to evaluate whether emotion recognition processing was associated with the involvement of the sensorimotor system in healthy participants and whether an alternative network was recruited in MBS participants.

Regarding the behavioural performance, we did not observe differences in accuracy for facial expressions versus animal shapes, both in healthy Control participants and in MBS participants. However, in terms of speed (i.e. RTs), Controls and MBS showed opposite patterns, with Control participants faster with facial expression stimuli than animal shapes stimuli, and MBS, vice versa, faster with animal shapes stimuli than with facial expression stimuli. Interestingly, a similar effect in terms of RTs was recently observed in individuals with temporarily acquired facial palsy (i.e. Bell's palsy; [76]).

To characterize the spatio-temporal unfolding of facial expression processing in both healthy Control and MBS participants, we employed cluster-based and permutation tests on ERPs and brain source signals, respectively.

In Control participants, testing for two early face-sensitive effects (facial expressions versus animal shapes) compatible with the temporal windows encompassing the P1 and the N170, the cluster-based permutation test revealed a difference between facial expressions and animal shapes that approached significance (*p* = 0.051) for the first temporal window and a significant difference for the second temporal window comprising the N170. For the P1 time range, the difference was most pronounced over occipito-parietal sensors, while in the N170 time range, the difference was characterized by two main clusters of activity, namely one negative cluster encompassing the N170 (which indicated a larger N170 for facial expressions than for animal shapes; see also [77]) and one positive cluster most pronounced from centro-parietal to frontal regions. The source-level analysis contrasting facial expressions versus animal shapes within the second temporal window (that included the more anterior positive cluster) indicated facial expression-selective recruitment of the right pSTS (a region critical within the core system and mainly involved in the processing of mutable aspects of faces such as facial expressions and eye gaze direction; [1]) and the right angular gyrus. Most interestingly for our aims, we observed selective recruitment of the left premotor cortex, the pars opercularis, orbitalis and triangularis of the left IFG, the left SMA, the left anterior cingulate cortex, and the medial portion of the OFC.

Precisely to account for the results converging with the simulation account, the latest version of the distributed neural model of face processing by Haxby & Gobbini [1] has included a motor simulation mechanism as a component of the extended system comprising the frontal operculum, aimed at assigning meaning in terms of emotion to facial expressions firstly visually analysed/detected in the core system, with a key role of the pSTS.

In brief, these results on healthy individuals dovetail nicely with the previous evidence supporting simulation and those results that provide evidence for a common neural basis for facial expressions observation and production [25–29]. Especially the IFG, in particular the operculum (BA 44), is conceived as a portion of an ancient system underlying emotional contagion recruited during emotion recognition and empathy tasks [78,79]. The left lateralization we have observed in ERP and source analyses could be interpreted as in line with the Arousal Hypothesis, which states that low arousing facial expressions of emotions tend to preferentially activate the left hemisphere, especially the left frontal operculum [78]. Here most of the facial expressions that participants viewed were of low intensity (i.e. low arousing) since they were extracted from morphing continua and, along with the Arousal Hypothesis, could have preferentially recruited a left neural pathway. The observation of the recruitment of the medial portion of the OFC deserves separate consideration. For a long time, OFC has been considered a region supporting cross-category and abstract general-domain processing of stimuli reward value, regardless of whether such stimuli are social or not (i.e. ‘common currency’ response in the OFC; [80]). However, more recently, some influential studies have provided evidence in support of the existence of face-selective patches in the OFC [81,82], in particular in the most medial portion, underlying affective and motivational face processing, and, in monkeys, preferentially responding to emotions and social categories, in particular, facial expressions, juvenile and female monkeys [81] in a time-window of around 130–220 ms following the face onset [81,82]. Since we did not match facial expressions and animal shapes for their motivational value, we cannot directly disentangle between the ‘common currency’ view and the face-selective proposal regarding the OFC activation we observed. However, the selective recruitment of the medial portion of OFC fits nicely with the latter view [82].

Interestingly, in MBS participants, the neural activity indicated a sensitivity to facial expressions (versus animal shapes) that occurred later in time from around 180 ms post-stimulus onset. We individuated two different temporal windows of facial expression sensitivity, namely between 180 and 250 ms and between 250 and 330 ms. The first of the two temporal windows was characterized by a negative cluster slightly right-lateralized at occipito-temporal sensors. This cluster is functionally compatible with an N170-like activity, although temporally delayed, of larger amplitude for facial expressions than animal shapes. The second, positive cluster indicated a significant difference between facial expressions and animal shapes that was most pronounced at the right centro-frontal sensors.

The source-level analysis (facial expressions versus animal shapes) within this averaged later temporal window showed the facial expression-selective recruitment of the right anterior inferior and middle temporal gyrus, the right temporopolar region, and the lateral OFC.

We interpret these whole findings in the light of degeneracy and neuroplasticity, while also showing that healthy individuals recruit regions critical for sensorimotor simulation during face processing.

Expanding from Haxby and Gobbini's [1] distributed neural model of face perception, more recently Duchaine and Yovel [83] proposed a revised neural framework of face processing to account for new evidence partially challenging the original model. To our aims, we want to stress here that in this revised model, both the fusiform face area (FFA) and pSTS contribute to facial expression processing, with the former being generically sensitive to *form information* (independent of this conveying emotional information) and pSTS selectively sensitive to *facial expressions* (see, e.g. [84]). Furthermore, the model integrates the discovery of additional face-selective areas by proposing the existence of two different processing pathways dedicated to face processing, namely a *ventral pathway* (including the anterior temporal lobe face area, ATL-FA) which preferentially represents form information but which nonetheless contributes to the processing of emotional expressions (see, e.g. [85]) and a *dorsal pathway* (which includes pSTS and the inferior frontal gyrus face area, IFG-FA), specialized in representing changing aspects of faces, such as expressions, direction of the gaze and movements of the mouth. Interestingly, Pitcher & Ungerleider [86] recently proposed a pathway of visual processing specialized for social perception. Notably, this pathway largely overlaps with the dorsal pathway of face processing of the model by Duchaine and Yovel [83]. The main common nodes between the dorsal pathway for face processing and the pathway for social perception are the pSTS face area (i.e. pSTS-FA) and the anterior STS face area (i.e. aSTS-FA), both selectively recruited in healthy participants in the present study. Conversely, a region compatible with the ATL-FA was selectively activated in MBS participants. As this is a central node for the ventral face processing pathway in the Duchaine and Yovel's [83] model, the conclusion of a degenerate and compensatory processing pathway in MBS appears even more plausible.^2^

One source of evidence in favour that both FFA and pSTS may play a role in expression recognition by extracting different sources of information from faces comes from a study by Said *et al*. [84], who presented computer-generated faces varying along two different dimensions, namely expression and typicality relative to the average face. These authors observed that FFA was sensitive to deviations for both dimensions while pSTS was sensitive to deviations from the average face in terms of expression. This pattern of findings may indicate that FFA is overall sensitive to even subtle differences in shape, and MBS individuals may use this information, at least for static subtle facial expressions, activating the ventral pathway when processing emotional faces. This is even more plausible when considering that the ATL-FA, which belongs to the ventral pathway and has direct connections with FFA, was selectively recruited in MBS participants. ATL-FA has been associated with tasks involving configural and/or identity face processing. Nevertheless, it is interesting to note that configural processing is important not only for face recognition but also for facial expression recognition because of the peculiar spatial arrangement of the features that characterize the different facial expressions and the different instances of the same facial expression (e.g. [88]).

In conclusion, our results seem to support the notion that in healthy individuals the preferential route for facial expression processing is the one involving the dorsal pathway and the motor system. At the same time, our findings suggest that the ventral pathway can contribute (at least for static emotional expressions) by the mechanisms of degeneracy and neuroplasticity, thus allowing normotypical behavioural performance in MBS individuals. We deem that the neural activity selectively observed in MBS is a marker of compensatory processes, also in the light of behavioural performance, which in terms of accuracy of facial expressions discrimination was similar to that of Controls (see, e.g. [46]). To note, however, the neural analysis revealed a delay of several tens of milliseconds in response selectivity to emotional faces in MBS participants compared to control participants. This delay could obviously have a noticeable dysfunctional impact in face-to-face interactions that future research needs to explore.

The possible advantage(s) in functional terms of reactivating sensorimotor representations in healthy individuals remain to be understood. One first possible advantage comes from experimental evidence suggesting that the dorsal/simulation pathway could be particularly efficient for face processing in poor/no-visibility conditions. For instance, Jiang *et al.* [89] investigated the processing time-course of faces with neutral and fearful expressions under conditions of interocular suppression and demonstrated that invisible fearful faces selectively recruit STS. Along the same line, emotional contagion and spontaneous mimicry have also been reported for unattended or unconscious facial expressions, thus advocating for their critical role in non-conscious emotion recognition (e.g. [90,91]). A second potential advantage concerns the possibility that motor simulation might bolster synchronization during social interactions. Facial mimicry, as a manifestation of motor simulation, is one of the primary mediators of emotional contagion; as such, facial mimicry is plausibly one of the main mechanisms of emotion and mood sharing, favouring coordination and synchronization between interacting individuals, both human and non-human animals, such as geladas, gorillas and dogs (see, e.g. [92]). Finally, the recruitment of one's motor system could make us ‘participants’ of others’ emotions rather than mere ‘spectators’ [93,94], allowing us to ‘feel’ others' emotions and enriching the subjective experience of the social world.

## Data Availability

The dataset and analyses reported in this article are available at Open Science Framework repository: osf.io/krpfb [49].

## Footnotes

1 Note, however, our own population statistical analyses on the original open data showed that in three out of five experiments MBS patients performed worse than Control participants. Generalized linear mixed models (i.e. logistic regression) were performed for experiments 1–5. The statistical model was the same across experiments: Score∼Group+(1|ID) (in Wilkinson notation). Score is the response variable (0 = wrong, 1 = correct), Group is a binary factor (MBS vs. Control participants) inserted as fixed effect and a random intercept for ID (subjects’ identifier) for the repeated measure design. For Experiment 1, *p* = .011; for Experiment 2, *p* < .001; for Experiment 3, *p* = .79; for Experiment 4, *p* = .038; and for Experiment 5, *p* = .121.

2 To note, the dorsal and ventral pathways of Duchaine & Yovel's [83] model do not correspond anatomically to those of the vision of the ‘two-stream hypothesis’ [87]. On the contrary, Pitcher & Ungerleider [86], when citing the dorsal and the ventral pathways, strictly refer to the ‘two-stream hypothesis’.
